# Variability, but Not Concentration, of Neuromelanin in the Substantia Nigra Is Associated With Individual Differences in Interference Control

**DOI:** 10.1002/hbm.70663

**Published:** 2026-11-09

**Authors:** Shuhui Lyu, Hongchi Zhang, Jimin Yan, Jiaqi Gao, Kangcheng Wang, Lorenza Colzato, Bernhard Hommel

**Affiliations:** ^1^ Shandong Provincial Key Laboratory of Brain Science and Mental Health, Faculty of Psychology Shandong Normal University Jinan China

**Keywords:** cognitive control, dopaminergic system, individual differences, interference control, neuromelanin‐sensitive MRI, substantia nigra

## Abstract

Interference control—the ability to suppress distracting information while selecting task‐relevant responses—relies on dopaminergic modulation of fronto‐striatal circuits. Although pharmacological evidence shows that transient dopaminergic enhancement can reduce interference, it remains unclear whether stable, trait‐like properties of the dopaminergic system constrain individual differences in control efficiency. The present study examined whether the concentration of the neuromelanin signal (NM‐Mean), and/or its variability, indexed by the standard deviation of the neuromelanin‐sensitive MRI contrast‐to‐noise ratio (NM‐SD), within the substantia nigra is associated with performance in the Flanker conflict task. Sixty healthy young adults completed the task. Neuromelanin CNR maps were registered to MNI152 standard space for ROI definition, and voxel‐wise CNR values within the SN–VTA region were extracted in native space to derive NM‐Mean and NM‐SD. NM‐Mean, indexing overall neuromelanin concentration, was not related to behavioral performance. In contrast, lower NM‐SD, reflecting more homogeneous dopaminergic integrity, was associated with smaller Flanker interference effects but was unrelated to conflict adaptation across trials. Statistically controlling for voxel size rendered the effect size no longer significant, but a trend remained. These findings provide limited and preliminary evidence that individual differences in dopaminergic signal reliability, rather than overall dopamine quantity, contribute to variability in cognitive control. Although NM‐SD is sensitive to acquisition‐related factors such as spatial resolution, the observed attenuation of its association with interference control after controlling for voxel size suggests that its behavioral relevance may reflect a mixture of biological variability and methodological influences rather than a clear trait‐like property of the dopaminergic system.

## Key Points

- Individual differences in neuromelanin signal heterogeneity (NM‐SD), but not overall neuromelanin signal (NM‐Mean), within the substantia nigra are associated with interference control across an independent EEG session, suggesting a stable, trait‐like association with dopaminergic integrity.
- NM‐SD may index inter‐individual differences in dopaminergic signal variability; however, its association with interference control was attenuated after controlling for voxel size and showed limited statistical evidence, indicating that NM‐SD estimates likely reflect a mixture of biological variability and acquisition‐related factors such as spatial resolution.

## Graphical abstract

Differences in neuromelanin signal heterogeneity (NM‐SD), not mean levels, predict interference control across sessions, suggesting a stable dopaminergic trait. However, this relationship weakens after accounting for voxel size, implying NM‐SD reflects both biological variability and imaging‐related factors.

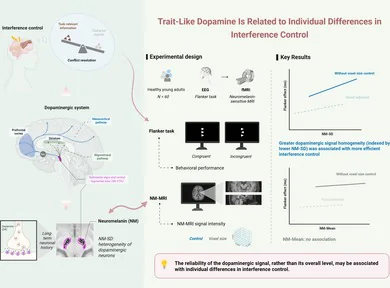

## 1 Introduction

Interference control—the ability to selectively enhance task‐relevant information while suppressing competing distractor signals—is essential for flexible behavior (Friedman and Miyake 2004). Performance in the Flanker task provides a clear example of this capacity, as successfully resolving conflict between target and distractor stimuli requires selecting the correct response while suppressing interference from competing information. This regulatory process is supported by dopaminergic modulation of fronto‐striatal circuits, which coordinate conflict monitoring, response selection, and the precision of attentional gating (Cools 2015; Frank 2005, 2025). By increasing the signal‐to‐noise ratio within these circuits, dopamine stabilizes task‐relevant representations while suppressing irrelevant input, thereby enhancing interference control (Li and Rieckmann 2014; Servan‐Schreiber et al. 1990).

Pharmacological evidence supports a causal role of dopamine in interference control. Administration of dopaminergic agents such as methylphenidate (MPH) reliably reduces Flanker interference effects and improves response efficiency (Gao et al. 2024; Koyun et al. 2025). Because MPH transiently increases extracellular dopamine by blocking dopamine (and noradrenaline) reuptake, these effects are commonly interpreted as phasic dopaminergic enhancements that sharpen control signals and reduce distractor influence on behavior. However, such manipulations primarily index short‐term changes in dopamine availability and do not capture stable properties of the dopaminergic system that may constrain interference control across individuals.

Contemporary dopamine theory distinguishes phasic dopamine signaling, which supports rapid adjustments to immediate task demands, from tonic, trait‐like dopaminergic properties that determine baseline network excitability and control stability (Badre 2025; Beeler et al. 2010; Mikhael et al. 2021). In the context of interference control, tonic dopaminergic integrity is thought to maintain stable gating of fronto‐striatal circuits, supporting sustained suppression of competing stimuli. Individuals with more reliable tonic dopaminergic signaling should therefore exhibit more consistent and efficient interference suppression in conflict tasks such as the Flanker task.

Neuromelanin‐sensitive MRI (NM‐MRI) provides a noninvasive marker of tonic dopaminergic properties. Neuromelanin is a by‐product of dopamine metabolism that accumulates slowly over the lifespan in dopaminergic neurons of the substantia nigra pars compacta, reflecting long‐term neuronal history rather than moment‐to‐moment dopamine release (Cassidy et al. 2019; Trujillo et al. 2024). NM‐MRI signal intensity is widely interpreted as a marker of dopaminergic neuron density and health. The average neuromelanin signal (NM‐Mean) reflects overall neuromelanin content within the substantia nigra and is commonly taken as an index of the general level of dopaminergic integrity or neuron abundance. Beyond average signal intensity, the standard deviation of the neuromelanin signal (NM‐SD) across the substantia nigra provides information about the heterogeneity and stability of dopaminergic neurons. Low NM‐SD reflects a relatively homogeneous population of neurons, supporting consistent dopamine signaling, whereas high NM‐SD indicates uneven dopaminergic integrity, potentially arising from patchy degeneration, focal vulnerability, or dysregulated dopamine metabolism (Trujillo et al. 2024). Critically, NM‐SD captures variability in dopaminergic function not apparent from mean neuromelanin alone (Trujillo et al. 2024). Accordingly, we expected NM‐SD to be positively associated with Flanker interference effects, such that higher NM‐SD would be associated with poorer interference control (i.e., larger Flanker effects), whereas lower NM‐SD would be associated with more efficient performance (i.e., smaller Flanker effects). In contrast, NM‐Mean indexes the average neuromelanin signal within the substantia nigra, which is typically interpreted as reflecting overall dopaminergic neuron density or integrity. Unlike NM‐SD, it does not capture spatial variability in neuromelanin distribution and therefore provides limited information about the consistency or reliability of dopaminergic signaling across neurons. Given that the present hypotheses specifically target variability‐related properties of the dopaminergic system, NM‐Mean was not expected to systematically be associated with individual differences in Flanker performance. Specifically, elevated NM‐SD may reflect less reliable dopamine signaling and noisier modulation of fronto‐striatal circuits, whereas lower NM‐SD should support more stable suppression of distractors and improved performance. Accordingly, neuromelanin SD in the substantia nigra may serve as a biomarker of interference control, whereas NM‐Mean is expected to be behaviorally noninformative in this context. By emphasizing NM‐SD, this approach highlights the role of dopaminergic signaling reliability, rather than overall dopaminergic quantity, in shaping cognitive control, beyond transient phasic effects captured by pharmacological manipulations.

To test our predictions, our study comprised two sessions: an initial session to collect data in the Flanker task and a subsequent MRI session to assess neuromelanin levels under off‐task resting‐state conditions.^1^ We considered the Flanker effect proper, reflecting overall conflict interference, and conflict adaptation—the trial‐by‐trial adjustment of control following high‐conflict trials (Botvinick et al. 2004). Conflict adaptation (a.k.a. Gratton effect) is thought to reflect the flexible upregulation of cognitive control in response to recently experienced conflict, resulting in reduced interference on subsequent trials. Whereas the Flanker effect indexes the general efficiency with which distractors are suppressed, conflict adaptation provides insight into the dynamic regulation of control and the system's capacity to adjust to changing task demands (Botvinick et al. 2004). Examining both measures is particularly informative in the context of dopaminergic function. If tonic dopaminergic integrity supports stable gating of fronto‐striatal circuits, it may not only facilitate baseline interference suppression but also promote the reliable implementation of adaptive control following conflict. Assessing conflict adaptation therefore allows us to test whether the reliability of dopamine signaling is linked not only to overall control efficiency but also to the moment‐to‐moment flexibility of control adjustments, offering a more comprehensive characterization of dopaminergic contributions to interference control.

## 2 Materials and Methods

### 2.1 Participants

The initial EEG dataset comprised 160 undergraduate students (74 males; mean age = 19.79 years, SD = 1.17), recruited via campus advertisements. This dataset and the design of the entire project, which includes a broader range of behavioral and individual‐difference measures, are publicly available on the Open Science Framework (OSF; https://osf.io/5j4e2/). A subset of 82 participants was randomly selected from this cohort to participate in the MRI session, which is also documented on OSF (https://osf.io/q4fvk/overview). The required sample size was determined using G*Power (version 3.1.9.7), with an alpha level of 0.05, a power of 0.95, and an expected effect size of *f* = 0.25, the calculation indicated that a minimum of 36 participants would be needed. Participants were excluded based on the following criteria: (1) incomplete neuromelanin‐sensitive MRI data due to technical issues (*n* = 20); (2) behavioral accuracy below chance level (< 50%, *n* = 1); and (3) insufficient image quality for ROI‐based analysis, as determined by visual inspection (*n* = 1). A participant flow diagram summarizing the selection and exclusion procedures is provided in Figure S1. The final sample consisted of 60 participants (25 males; mean age = 20.42 years, SD = 1.06, see Table 1). Comparisons between included and excluded participants revealed no significant differences in demographic characteristics (age and sex) or available behavioral measures (interference effects, accuracy, and reaction times; all *p* > 0.10; Tables S1 and S2).

**TABLE 1:** Demographic, behavioral, and MRI characteristics of the final participants (*N* = 60).

Features	Mean	SD	Range
Gender (male/female)	25/35	—	—
Age (years)	20.42	1.06	19.00–22.00
Reaction time (ms)			
FE	59.47	28.46	−39.00 to 106.00
GE	15.47	22.64	−35.22 to 69.73
RT_cC_	300.29	20.64	255.86–343.96
RT_cI_	369.11	22.45	261.18–399.94
RT_iC_	309.24	21.09	265.58–357.00
RT_iI_	362.59	18.86	304.74–393.90
Neuromelanin			
NM‐Mean	0.19	0.04	0.10–0.31
NM‐SD	0.19	0.02	0.13–0.25

*Note:* Descriptive statistics for the 60 participants included in the final analysis. For continuous variables, values represent the mean and standard deviation (SD), along with the range (minimum – maximum). Counts are provided for categorical variables (Gender). Age is reported in years; all reaction times (RT) and Flanker effects (FE) are reported in milliseconds (ms). FE represents the conflict interference cost, calculated as RT_Incongruent_ – RT_Congruent_. RT sequences for the conflict adaptation (GE, Gratton effects) analysis are defined by the congruency of the previous and current trials: RT_cC_ (congruent–congruent), RT_cI_ (congruent–incongruent), RT_iC_ (incongruent–congruent), and RT_iI_ (incongruent–incongruent). Neuromelanin‐sensitive MRI (NM‐MRI) metrics were quantified using the contrast‐to‐noise ratio (NM‐CNR) within the SN–VTA region of interest. NM‐Mean and NM‐SD denote the mean and standard deviation of voxel‐wise NM‐CNR values within the ROI.

All participants were right‐handed, had normal or corrected‐to‐normal vision, and reported no personal or family history of psychiatric or neurological disorders. Written informed consent was obtained prior to participation. Participants received monetary compensation of 400 CNY upon completion of both experimental sessions (200 CNY per session). All study procedures were approved by the Ethics Committee of the School of Psychology, Shandong Normal University.

### 2.2 Experimental Design and Procedure

The study comprised an initial behavioral session and a subsequent MRI session several months later. The primary aim was to test whether individual differences in the structural integrity of the nigral dopaminergic system, indexed by neuromelanin‐sensitive MRI (NM‐MRI), are associated with conflict interference as measured by the Flanker effect and conflict adaptation in a flanker task.

Behavioral performance was assessed using the Flanker task paradigm. Although NM‐MRI data were acquired at a later time point, neuromelanin signals are considered a relatively stable marker of dopaminergic neuronal integrity in healthy young adults, reflecting long‐term accumulation rather than short‐term functional fluctuations (Cassidy et al. 2019; Zecca et al. 2004). Accordingly, NM‐MRI measures were treated as trait‐level indices of nigral structural integrity in all subsequent analyses.

#### 2.2.1 Flanker Task

Participants performed a modified Flanker task adapted from previous studies (Konjusha et al. 2022; Mückschel et al. 2017; Pscherer et al. 2020); the timing of events is shown in Figure 1C. The task comprised four blocks of 120 trials each (480 trials in total). Each trial began with a central fixation cross presented for a jittered interval of 700–1000 ms, followed by a cue stimulus consisting of two vertically aligned triangles presented for 200 ms. Subsequently, a central target triangle appeared between the cue stimuli for 300 ms. After target offset, a response window remained until a response was made or until a randomly jittered interval of 700–1000 ms had elapsed.

**FIGURE 1:**
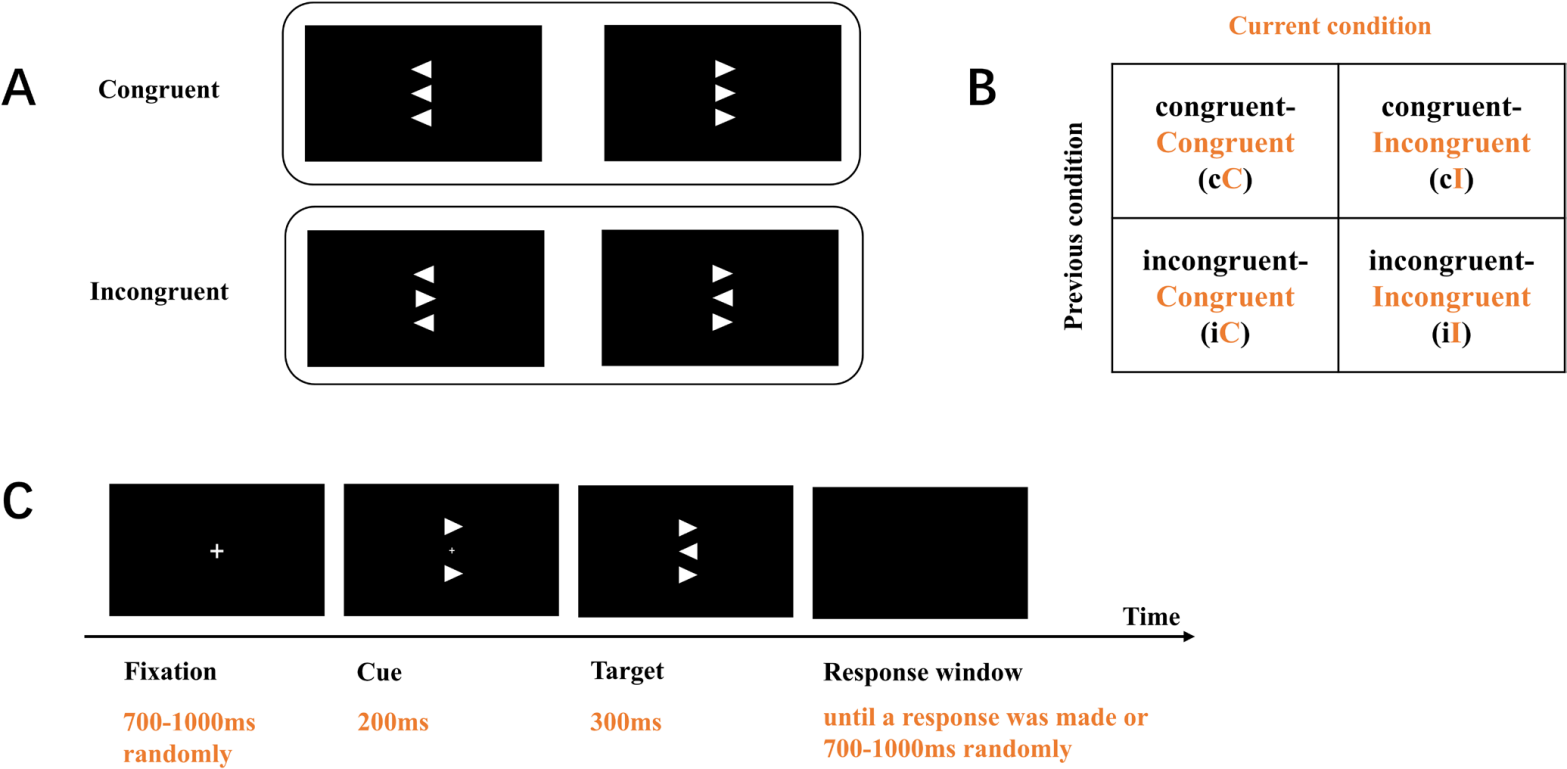
Task design and trial structure. (A) Task conditions. In congruent trials, the flanking stimuli pointed in the same direction as the central target; in incongruent trials, the flankers pointed in the opposite direction, thereby inducing response conflict. Participants were instructed to respond as quickly and accurately as possible to the direction of the central target (see Section 2 for details). (B) Trial sequence categorization for the conflict adaptation (Gratton) analysis. Trials were classified according to the congruency of the previous and current trials: congruent–congruent (cC), congruent–incongruent (cI), incongruent–congruent (iC), and incongruent–incongruent (iI). (C) Trial structure. Each trial began with a fixation cross, followed by a cue stimulus and a centrally presented target requiring a left or right response. A response window followed target offset.

Trials were classified as congruent or incongruent depending on whether the flanking triangles pointed in the same or opposite direction as the central target (Figure 1A). To establish a dominant response tendency and enhance reactive conflict processing, congruent, and incongruent trials were presented in a 2:1 ratio (320 congruent, 160 incongruent), with both conditions balanced across left‐ and right‐pointing target directions. Participants were instructed to respond as quickly and accurately as possible using their right hand, pressing the “J” key for right‐pointing targets and the “F” key for left‐pointing targets. Prior to the main experiment, participants completed a practice block of 20 trials to ensure task comprehension.

#### 2.2.2 Behavioral Data Analysis

Behavioral analyses focused on reaction time (RT) measures. Only correct‐response trials were included in all analyses. To quantify trial‐by‐trial adjustment in cognitive control, we calculated the Gratton effect (sequential modulation of conflict; Gratton et al. 1992). As illustrated in Figure 1B, trials were categorized into four sequence types based on the congruency of the previous (*n* − 1) and current (*n*) trials: congruent–congruent (cC), congruent–incongruent (cI), incongruent–congruent (iC), and incongruent–incongruent (iI). Mean RTs were computed separately for each sequence for each participant. Only trials with correct responses on both the current and preceding trial were included, and the first trial of each block was excluded because it lacked a preceding trial. In addition, conflict processing efficiency was indexed by the Flanker interference effect (RT_Incongruent_ − RT_Congruent_). Sequential conflict adaptation (Gratton effect) was assessed at the group level by the interaction between previous‐ and current‐trial congruency. For analyses of individual differences, a subject‐specific Gratton effect index was additionally computed using a difference score: (RT_cI_ − RT_cC_) − (RT_iI_ − RT_iC_), reflecting the reduction of interference following incongruent relative to congruent trials.

### 2.3 MRI Acquisition and Preprocessing

All MRI data were acquired on a 3 T Siemens Prisma scanner (Siemens Healthineers, Erlangen, Germany) using a 64‐channel head coil. Foam padding was applied to minimize head motion during scanning.

Neuromelanin‐sensitive (NM‐MRI) images were acquired using a high‐resolution 2D T1‐weighted spoiled gradient‐recalled echo (FLASH) sequence with magnetization transfer contrast (MTC) preparation (TR = 1100 ms, TE = 3.9 ms, flip angle = 40°, slice thickness = 1 mm with no inter‐slice gap, 64 slices, bandwidth = 340 Hz/pixel). Two signal averages (NEX = 2) were acquired, with the repeated acquisitions averaged in k‐space prior to Fourier reconstruction, resulting in a single reconstructed NM‐MRI image. Because the two signal averages were combined before image reconstruction, they did not yield two separately reconstructed NM‐MRI volumes. Consequently, retrospective realignment between individual averages and the extraction of conventional inter‐volume motion parameters were not possible. To ensure compliance with specific absorption rate (SAR) limits and optimize signal acquisition at the individual level, the in‐plane field of view (FOV) was automatically determined by the scanner's prescan calibration procedures. This hardware‐driven adjustment resulted in five distinct in‐plane voxel sizes across participants (0.51 × 0.51 mm^2^, 0.55 × 0.55 mm^2^, 0.59 × 0.59 mm^2^, 0.63 × 0.63 mm^2^, and 0.66 × 0.66 mm^2^), while maintaining a constant slice thickness of 1 mm. The total acquisition time for the NM‐MRI sequence was approximately 10 min 53 s. Image stacks were aligned parallel to the anterior commissure–posterior commissure (AC–PC) line, ensuring coverage of the substantia nigra/ventral tegmental area (SN/VTA) and adjacent brainstem regions (Cassidy et al. 2019; Salzman et al. 2021). All reconstructed NM‐MRI images underwent immediate visual quality inspection by two independent raters. Participants were instructed to remain still throughout the acquisition, and foam padding was used to minimize head motion. Scans showing substantial motion‐related degradation were reacquired when feasible. This quality‐control procedure was designed to identify and mitigate visually apparent motion artifacts, although subtle within‐acquisition motion could not be retrospectively quantified from the final reconstructed image. Scans with substantial motion‐related artifacts were reacquired when possible. A total of 62 participants successfully completed the MRI acquisition. One dataset was later excluded due to insufficient image quality for ROI‐based analysis.

For anatomical reference and spatial registration, a high‐resolution three‐dimensional T1‐weighted structural image was acquired using a SPACE (Sampling Perfection with Application optimized Contrast using different flip angle Evolutions) sequence with the following parameters: TR = 2400 ms, TE = 3.47 ms, inversion time (TI) = 1000 ms, flip angle = 8°, matrix = 320 × 320, 208 slices, and voxel resolution = 0.8 × 0.8 × 0.9 mm^3^. Total acquisition time for the T1‐weighted sequence was approximately 5 min 33 s.

MRI data preprocessing and analysis were conducted using a pipeline integrating FSL (v6.0.7.14), FreeSurfer (v7.4.1) and Advanced Normalization Tools (ANTs, v2.5.4), adapted from the workflow described by Vano et al. (2024). Briefly, preprocessing included: (1) NM‐MRI images were first cropped using FSL RobustFOV to remove nonbrain regions, followed by intensity nonuniformity correction using FreeSurfer's mri_nu_correct.mni; (2) rigid‐body co‐registration of each participant's bias‐corrected NM‐MRI image to the corresponding individual T1‐weighted image using ANTs; and (3) nonlinear normalization of the T1‐weighted image to the Montreal Neurological Institute (MNI) 152 template (Mazziotta et al. 1995) using an ANTs multistage registration procedure consisting of rigid‐body, affine, and symmetric diffeomorphic (SyN) transformations. The resulting transformation parameters were applied in both forward and inverse directions. Forward transformations were used to normalize individual NM‐derived CNR maps into MNI152 space for subsequent group‐level spatial analyses, whereas inverse transformations were used to warp atlas‐defined SN–VTA and CC masks into each participant's native NM‐MRI space for individual‐level NM‐CNR quantification.

### 2.4 MRI Data Analysis

Regions of interest (ROIs) were defined using the neuromelanin‐sensitive MNI152 template and atlas‐derived labels of the substantia nigra/ventral tegmental area (SN–VTA) and crus cerebri (CC) developed by Vano et al. (2024). To facilitate signal quantification in native space, the SN‐VTA (target) and CC (reference) masks were back‐projected from MNI space into each participant's native NM‐MRI space by applying the inverse of the previously computed nonlinear warp fields and affine transformation using ANTs. Registration accuracy was visually assessed by inspecting the alignment between back‐projected ROIs and bias‐corrected NM‐MRI images. The atlas boundaries showed good anatomical correspondence with neuromelanin‐related hyperintensity in the midbrain across participants, see Figure 2.

**FIGURE 2:**
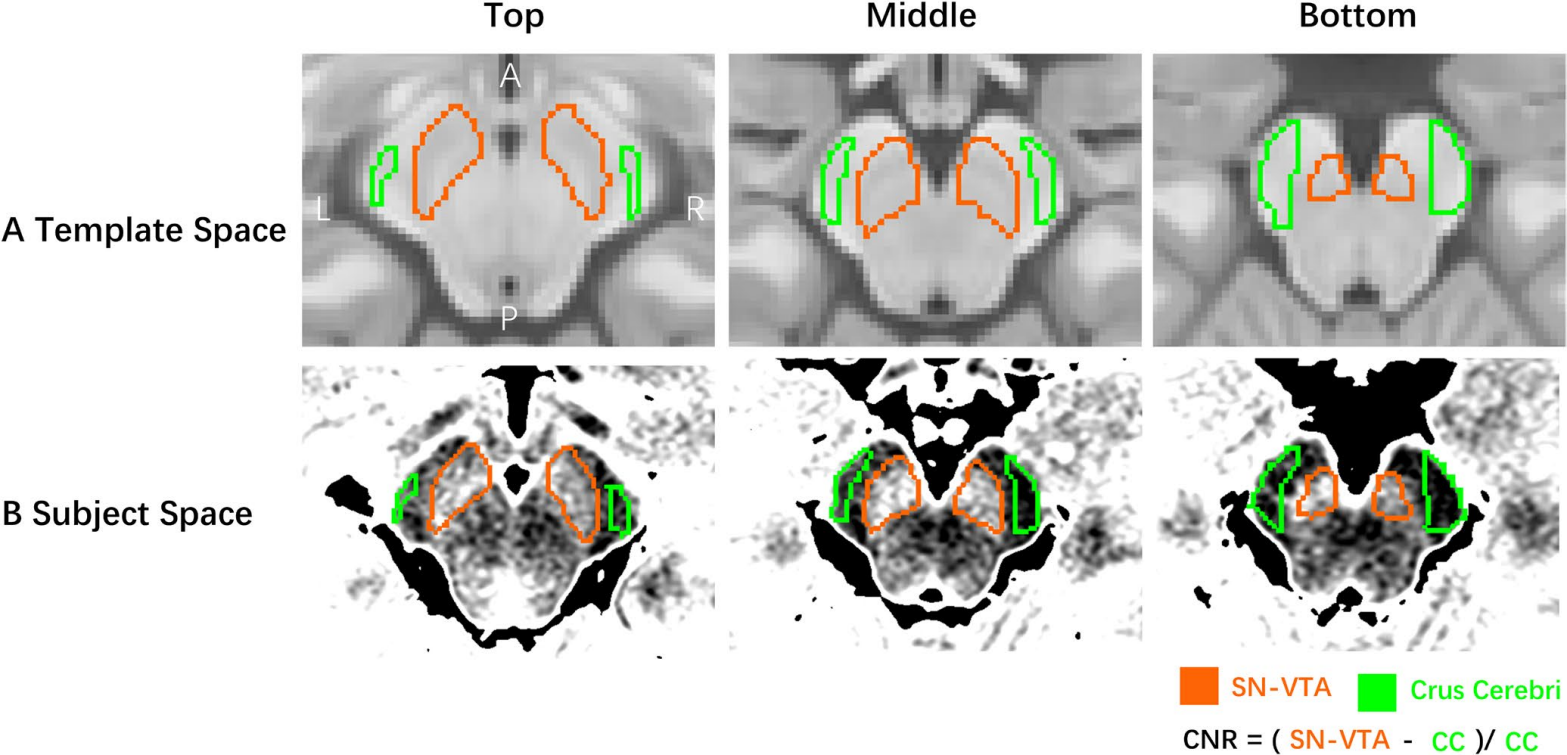
Definition and transformation of neuromelanin ROIs. (A) SN–VTA target region (orange) and crus cerebri (CC) reference region (green) defined in MNI template space using probabilistic atlases. (B) Corresponding ROIs back‐projected into the native NM‐MRI space of a representative participant, overlaid on bias‐corrected images. Images are displayed in neurological convention (left hemisphere shown on the left). A, anterior; P, posterior; L, left; R, right.

To account for inter‐subject variability in absolute MRI signal intensity, NM signal was quantified using the contrast‐to‐noise ratio (CNR). The CC was selected as the reference region due to its anatomical proximity and absence of neuromelanin (Cassidy et al. 2019). Voxel‐wise NM‐CNR maps were calculated in native space using the follows:

$$\mathrm{NM}-\mathrm{CNR}=\frac{I_{\mathrm{SN}-\mathrm{VTA}}-{\text{Mode}}_{\mathrm{cc}}}{{\text{Mode}}_{\mathrm{cc}}}$$

where $I_{\mathrm{SN}–\mathrm{VTA}}$ denotes the signal intensity of each voxel within the SN–VTA, and $\mathrm{Mod}e_{\mathrm{cc}}$ represents the modal intensity of the crus cerebri reference region estimated using kernel density estimation.

For ROI‐based statistical analysis, both the mean (NM‐Mean) and standard deviation (NM‐SD) of voxel‐wise NM‐CNR values were computed within the bilateral SN–VTA mask in each participant's native space, ensuring preservation of individual signal characteristics. Specifically, these metrics were calculated across the combined left and right SN–VTA ROI rather than within each hemisphere separately. NM‐Mean reflects overall neuromelanin‐related signal intensity, whereas NM‐SD indexes spatial heterogeneity of the signal.

To characterize group‐level spatial organization, voxel‐wise statistics (including group mean and inter‐individual standard deviation maps) were computed in MNI152 standard space across all included participants (*N* = 60). Additionally, a hotspot probability map was generated to assess spatial consistency by identifying the top 20% highest NM‐CNR voxels for each individual and computing their overlap frequency across the cohort.

### 2.5 Multivariate Relationship Between NM Intensity and Gratton Effect

Statistical analyses were conducted using Python (version 3.12.4) with the *pandas* (v2.2.2), *scipy* (v1.13.1), *numpy* (v1.26.4), *pingouin*, and *statsmodels* libraries. To establish the presence of sequential conflict adaptation (Gratton effect), mean RTs were submitted to separate 2 × 2 repeated‐measures analyses of variance (ANOVAs), with Previous‐trial congruency (Congruent, Incongruent) and Current‐trial congruency (Congruent, Incongruent) as within‐subject factors. A significant interaction between previous‐ and current‐trial congruency was taken as evidence for conflict adaptation. When significant interactions were observed, follow‐up paired‐sample *t*‐tests were used to directly compare the magnitude of the Flanker interference effect (RT_Incongruent_ − RT_Congruent_) following incongruent versus congruent trials, thereby quantifying the reduction in interference following conflict. For individual‐differences analyses, overall conflict processing efficiency was quantified using the global Flanker interference effect, defined as the difference between reaction times in incongruent and congruent trials (RT_Incongruent_ − RT_Congruent_). Conflict adaptation was indexed by a subject‐specific Gratton effect, calculated as ((RT_cI_ − RT_cC_) − (RT_iI_ − RT_iC_)).

To test the primary hypothesis regarding the structural integrity of the nigral dopaminergic system, neuromelanin‐sensitive MRI (NM‐MRI) metrics derived from the SN–VTA were examined, including the mean contrast‐to‐noise ratio (NM‐Mean) and the spatial heterogeneity of the NM signal (NM‐SD). Given potential deviations from normality in imaging and behavioral measures, nonparametric permutation tests (5000 iterations) were used to estimate statistical significance.

Two complementary permutation‐based correlation analyses were conducted. First, zero‐order permutation tests were performed to assess unadjusted associations between NM‐MRI metrics and behavioral effects. Second, partial permutation tests based on the Freedman–Lane procedure were conducted to evaluate the robustness of these associations after accounting for potential acquisition‐related variability (Freedman and Lane 1983). Individual differences in voxel size (defined as Resolution_X × Resolution_Y × slice thickness) were included as a continuous covariate to avoid information loss associated with discretizing acquisition parameters and to preserve the original covariance structure of the data. The Freedman–Lane procedure permutes residuals after regressing out the covariate, thereby maintaining appropriate control of Type I error rates. Benjamini–Hochberg FDR correction was applied separately within two prespecified families: (a) four zero‐order correlation analyses and (b) four voxel‐size‐adjusted correlation analyses.

In parallel, Bayesian analyses were conducted using the same data and model specifications as the frequentist analyses. Specifically, Bayesian correlation was used for zero‐order associations, and Bayesian multiple linear regression was applied for covariate‐adjusted analyses, with behavioral effects specified as dependent variables and NM‐MRI metrics and voxel size entered as predictors. The inclusion Bayes factor (BF_inclusion_) quantified evidence for the unique contribution of NM‐MRI metrics after accounting for voxel size.

For frequentist analyses, *p*‐values were obtained from permutation distributions, and 95% confidence intervals (CIs) for both zero‐order and partial correlations were estimated via bootstrap resampling (5000 iterations). For Bayesian analyses, default priors were used, and evidence strength was interpreted according to standard conventions (Lee and Wagenmakers 2014), with BF_10_ > 3 indicating moderate evidence and BF_10_ < 1/3 indicating evidence in favor of the null hypothesis. In Bayesian linear regressions, inclusion Bayes factors (BF_inclusion_) were used to quantify evidence for the contribution of individual predictors, with values > 1 indicating support for inclusion and values < 1 indicating support for exclusion, and values close to 1 indicating limited or anecdotal evidence. Posterior inclusion probabilities (*P*(incl|data)) are reported as descriptive measures of variable importance. Bayesian analyses were conducted using JASP (v0.17.1.0), whereas permutation testing, bootstrapping, and visualization were implemented in Python using matplotlib (v3.8.4) and seaborn (v0.13.2).

A sensitivity analysis was additionally conducted using G*Power (version 3.1.9.7) to estimate the minimum detectable effect sizes for the correlation‐based analyses. With *N* = 60, *α* = 0.05 (two‐tailed), and power = 0.80, the minimum detectable effect size was |*r*| = 0.35 for zero‐order Pearson correlations. For the voxel‐size‐adjusted regression model including NM‐SD and voxel size as predictors, the minimum detectable effect size was *f* ^2^ = 0.135.

## 3 Result

### 3.1 Behavioral Performance and Conflict Adaptation

Descriptive statistics for behavioral performance are presented in Table 1. A 2 × 2 repeated‐measures ANOVA revealed a robust main effect of current‐trial congruency (*F*_(1,59)_ = 339.49, *p* < 0.001, $\eta_{p}^{2}$ = 0.85), reflecting the canonical Flanker effect, with slower responses on incongruent compared to congruent trials. Critically, as shown in Figure 3A, the analysis also showed a highly significant interaction between previous‐ and current‐trial congruency ($F_{\left(1,59\right)}$ = 28.00, *p* < 0.001, $\eta_{p}^{2}$ = 0.32), indicating a reliable sequential modulation of the Flanker interference effect, commonly referred to as the Gratton effect (Gratton et al. 1992). Follow‐up analyses focused on the interaction revealed a significant reduction of the Flanker effect following incongruent relative to congruent trials ($t_{\left(59\right)}$ = 5.29, *p* < 0.001, Cohen's *d* = 0.55; Figure 3B). Interference effects were robustly present under both trial‐history conditions (both *p* < 0.001), confirming reliable response conflict across conditions.

**FIGURE 3:**
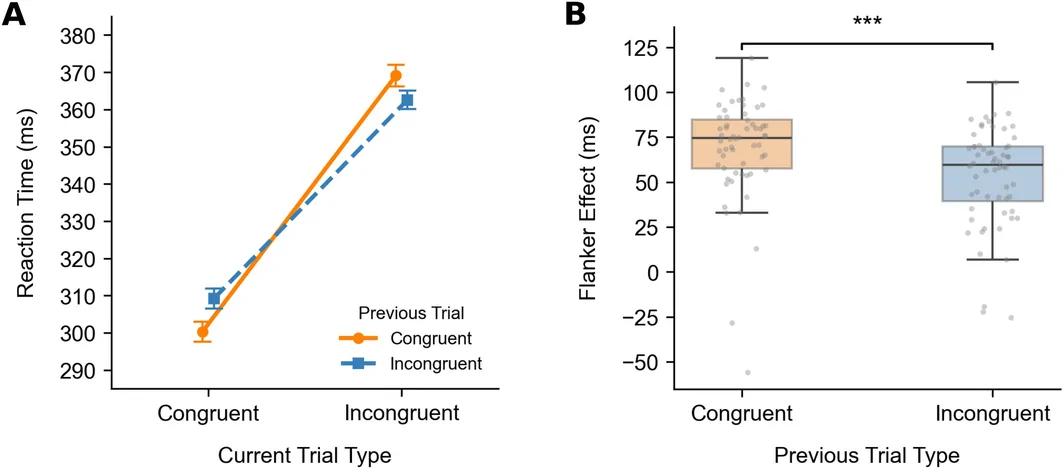
Behavioral validation of conflict adaptation. (A) Interaction plots depicting mean reaction times (RTs) as a function of previous‐ and current‐trial congruency. Error bars denote the standard error of the mean. (B) Raincloud plots illustrating the distribution of Flanker interference effects (RT_Incongruent_—RT_Congruent_) following congruent and incongruent trials. Each plot combines boxplots with individual participant data points. The Flanker interference effect was significantly smaller following incongruent compared to congruent trials, reflecting conflict adaptation (Gratton effect). ***p < 0.001.

### 3.2 Association Between SN–VTA Neuromelanin Signal Heterogeneity and Interference Control

#### 3.2.1 Spatial Characterization of SN–VTA Signal

Descriptive statistics for the subject‐level NM‐Mean and NM‐SD measures are presented in Table 1. To characterize the baseline spatial organization of the neuromelanin‐sensitive signal, voxel‐wise group statistics were computed within the SN–VTA mask (*N* = 60). The group mean NM‐CNR map showed a spatial gradient, with higher signal intensity observed in the ventrolateral substantia nigra pars compacta (SNc) and lower intensity toward surrounding regions (Figure 4A). Hotspot probability mapping, reflecting the inter‐subject spatial overlap of the top 20% highest NM‐CNR voxels, revealed a localized cluster within the ventrolateral SNc (Figure 4B). The group standard deviation (SD) map, indexing inter‐individual variability at the voxel level, showed a broader spatial distribution within the SN–VTA mask (Figure 4C). The spatial patterns observed in the mean, hotspot, and SD maps were not identical across voxels.

**FIGURE 4:**
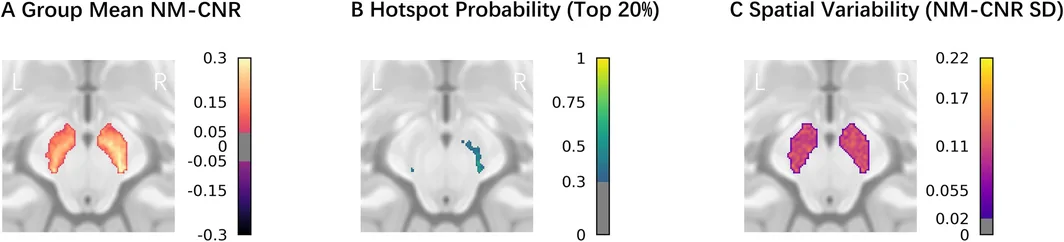
Spatial characterization and architectural variability of the neuromelanin‐sensitive signal within the SN‐VTA. Axial slices at *z* = −12 (MNI152 space) show voxel‐wise maps across participants (*N* = 60). (A) Group mean NM‐CNR map. (B) Hotspot probability map showing the overlap of the top 20% highest NM‐CNR voxels across subjects. (C) Group standard deviation (SD) map reflecting inter‐individual variability. L, left; R, right. Color bars indicate voxel values (NM‐CNR in A and C; proportion of subjects exhibiting top 20% NM‐CNR in B).

#### 3.2.2 ROI‐Based Association Analysis

We next examined the association between nigral structural heterogeneity (NM‐SD) and interference control using permutation‐based statistical analyses (5000 permutations). A positive zero‐order association was observed between NM‐SD and the Flanker interference effect (*r* = 0.321, *p* = 0.012; FDR adjusted *p* = 0.048; Figure 5A). Bayesian correlation provided moderate evidence for this relationship (BF10 = 3.393).

**FIGURE 5:**
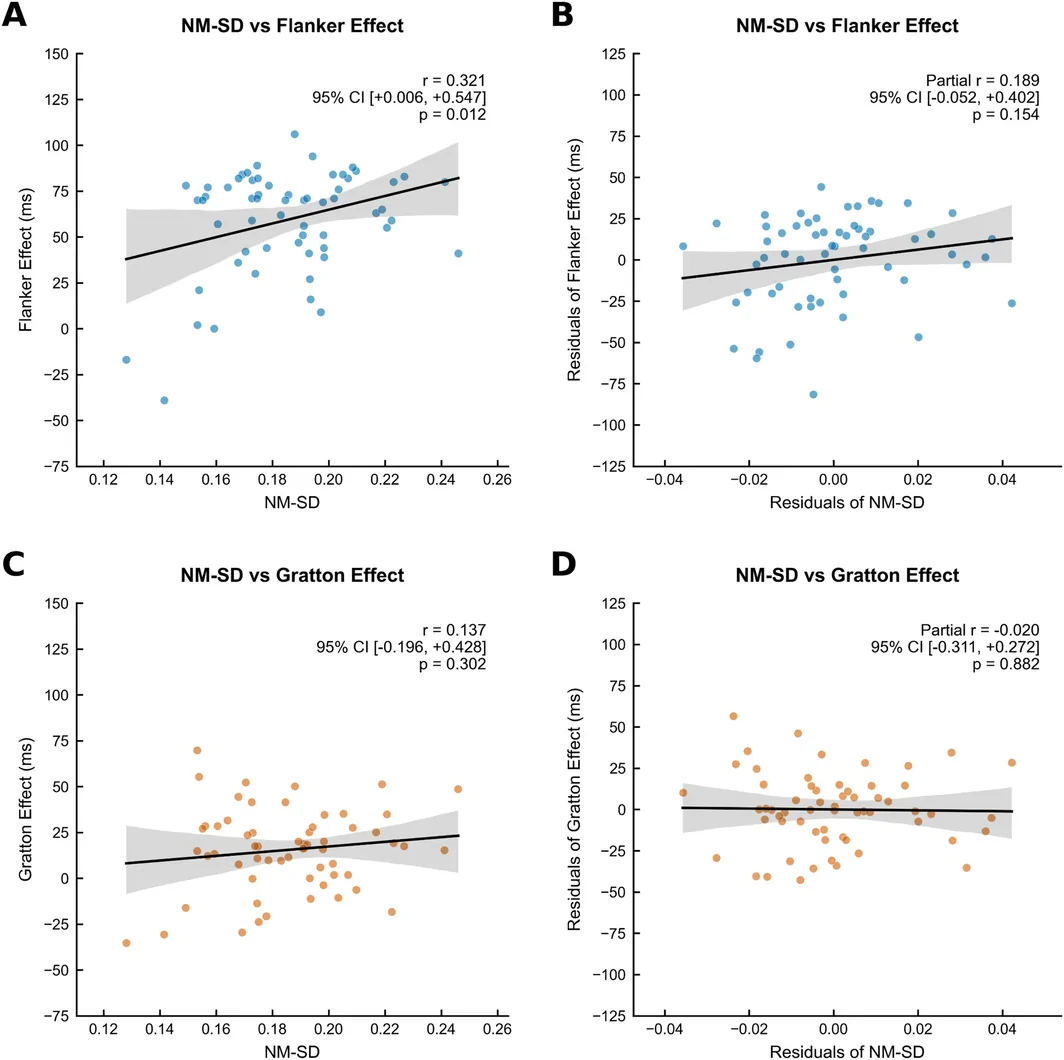
Association between nigral structural heterogeneity (NM‐SD) and behavioral indices of cognitive control. Scatter plots show the relationship between NM‐SD within the SN–VTA across participants (*N* = 60). (A and B) Zero‐order correlations between NM‐SD and the Flanker interference effect (A), and partial correlations controlling for voxel size (B). (C and D) Zero‐order correlations between NM‐SD and conflict adaptation (Gratton effect) (C), and partial correlations controlling for voxel size (D). In (B) and (D), axes represent residualized values after regressing out voxel size using the Freedman–Lane procedure. Each panel reports the correlation coefficient (*r*), bootstrapped 95% confidence interval (CI; 5000 resamples), and permutation‐based *p*‐value (p_perm). Shaded regions indicate the 95% CI of the fitted regression line.

Because voxel size varied across participants and may influence spatial variability metrics such as NM‐SD, we next evaluated whether the NM‐SD–Flanker association remained after adjustment for voxel size using the Freedman–Lane permutation procedure (Winkler et al. 2014). After controlling for voxel size, the association was attenuated (*r* = 0.189, *p* = 0.154; Figure 5B). Consistent with this, Bayesian linear regression indicated that, after adjusting for voxel size, evidence for including NM‐SD as a predictor was limited (BF_inclusion_ = 1.803, *P*(incl|data) = 0.643). Additional sensitivity analyses controlling for native‐space SN–VTA ROI voxel count yielded comparable results (Table S3).

We then examined the association between NM‐SD and conflict adaptation using permutation‐based statistical analyses (5000 permutations). As shown in Figure 5C, the correlation did not reach statistical significance (*r* = 0.137, *p* = 0.302), and Bayesian analysis provided evidence in favor of the null hypothesis (BF_10_ = 0.275). After controlling for voxel size, the association remained negligible (*r* = −0.020, *p* = 0.882; Figure 5D). Similarly, Bayesian linear regression indicated limited evidence for the inclusion of NM‐SD as a predictor (BF_inclusion_ = 0.350, *P*(incl|data) = 0.259). Bootstrapped 95% confidence intervals for all correlation coefficients are reported in Figure 5.

#### 3.2.3 Specificity Analysis

To assess specificity, we examined whether overall neuromelanin content (NM‐Mean) was associated with interference control. As shown in Figure 6, no significant associations were observed between NM‐Mean within the SN–VTA and the Flanker interference effect, either before or after controlling for voxel size. The zero‐order correlation was not significant (*r* = 0.060, *p* = 0.649; Figure 6A), and Bayesian analysis indicated limited evidence for an association (BF_10_ = 0.178). After controlling for voxel size, the partial correlation remained nonsignificant (*r* = 0.045, *p* = 0.724; Figure 6B). Consistent with this, Bayesian linear regression indicated limited evidence for including NM‐Mean in the model (BF_inclusion_ = 0.401, *P*(incl|data) = 0.286).

**FIGURE 6:**
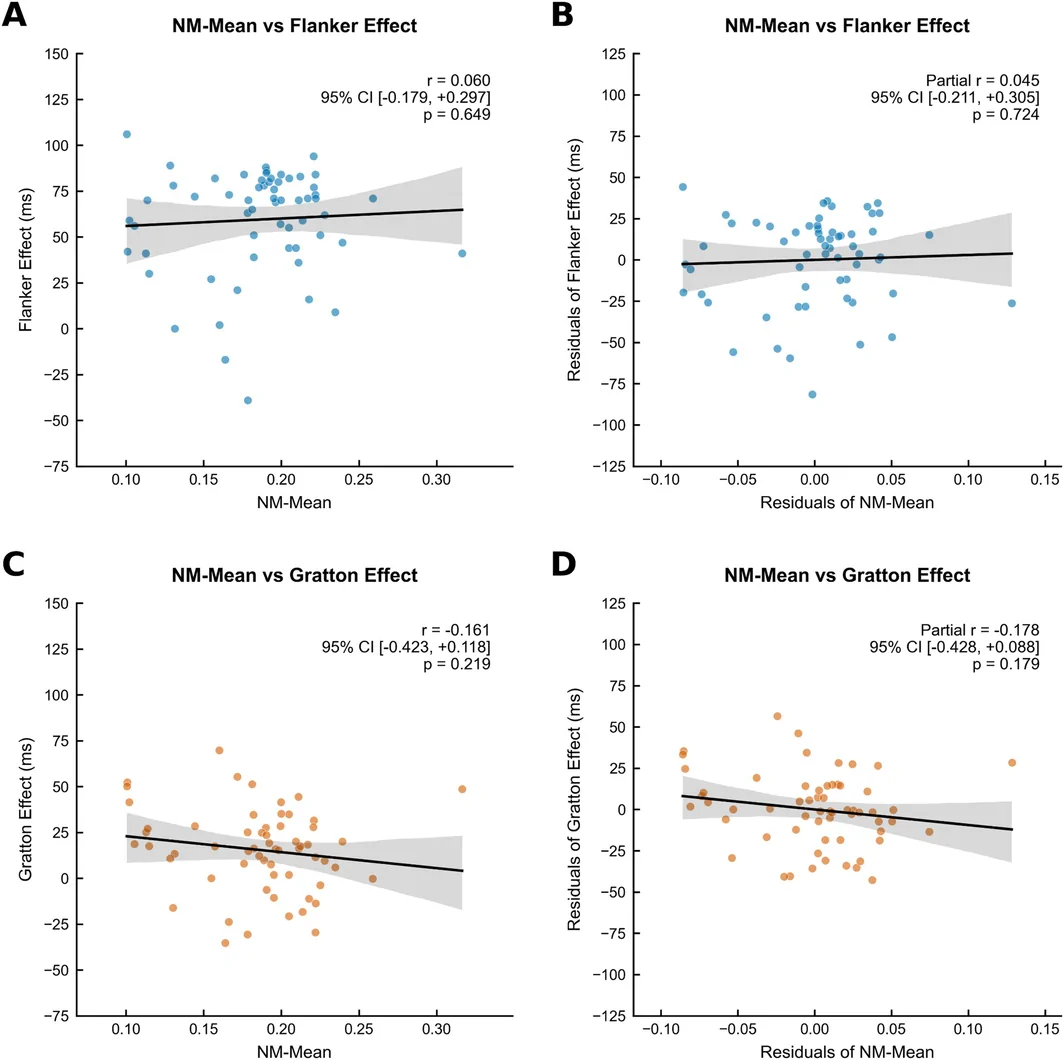
Association between overall neuromelanin content (NM‐Mean) and behavioral indices of cognitive control. Scatter plots show the relationship between NM‐Mean within the SN–VTA across participants (*N* = 60). (A and B) Zero‐order correlations between NM‐Mean and the Flanker interference effect (A), and partial correlations controlling for voxel size (B). (C and D) Zero‐order correlations between NM‐Mean and conflict adaptation (Gratton effect) (C), and partial correlations controlling for voxel size (D). In (B) and (D), axes represent residualized values after regressing out voxel size using the Freedman–Lane procedure. Each panel reports the correlation coefficient (*r*), bootstrapped 95% confidence interval (CI; 5000 resamples), and permutation‐based *p*‐value (p_perm). Shaded regions indicate the 95% CI of the fitted regression line.

We further examined the association between NM‐Mean and conflict adaptation. No significant associations were observed either before (*r* = −0.161, *p* = 0.219; Figure 6C) or after controlling for voxel size (*r* = −0.178, *p* = 0.179; Figure 6D). Bayesian analyses indicated limited evidence for an association at the zero‐order level (BF_10_ = 0.337) and similarly limited evidence for including NM‐Mean in the voxel‐size‐adjusted model (BF_inclusion_ = 0.599, *P*(incl|data) = 0.375). Across the two prespecified FDR correction families, the zero‐order NM‐SD–Flanker association was the only association that survived FDR correction (FDR adjusted *p* = 0.048); no other zero‐order or voxel‐size‐adjusted association survived correction (see Table S4 for full FDR results).

## 4 Discussion

The present study investigated whether stable properties of the dopaminergic system are associated with individual differences in interference control. By combining MN‐MRI with behavioral performance on the Flanker task, we found that variability in dopaminergic integrity—indexed by NM‐SD, but not NM‐Mean, within the substantia nigra—is meaningfully associated with the efficiency and reliability of conflict processing. NM‐Mean, reflecting overall neuromelanin signal and commonly interpreted as a proxy of general dopaminergic neuron abundance, was included to dissociate effects of dopaminergic quantity from variability in signal organization. Specifically, individuals exhibiting lower NM‐SD showed reduced interference and more consistent performance across sessions, whereas higher NM‐SD was linked to larger Flanker effects and diminished regulatory stability. The absence of effects for NM‐Mean suggests that overall dopaminergic integrity alone is insufficient to explain individual differences in cognitive control. Instead, findings support the view that the reliability of dopamine signaling, rather than dopaminergic quantity alone, plays a central role in shaping cognitive control. It is important to clarify that our interpretation of NM‐SD is conceptual rather than mechanistic. NM‐SD should not be understood as a direct measure of dopamine signaling reliability, but rather as a quantitative measure of the spatial dispersion of voxel‐wise neuromelanin‐sensitive CNR values within the SN–VTA ROI. The biological determinants of this dispersion have not yet been fully established, particularly in healthy young adults. Potential contributors include individual differences in nigral morphology and microstructure, the spatial distribution and density of neuromelanin‐containing tissue, iron content, tissue composition within the ROI, and partial‐volume effects. However, the existence of multiple possible contributors does not render NM‐SD biologically meaningless or prevent its behavioral relevance from being investigated. Rather, NM‐SD should currently be regarded as a composite imaging phenotype whose specific biological basis remains to be determined. The present findings provide preliminary evidence that individual differences in this spatial property of the NM‐MRI signal may be associated with interference control, while not establishing the mechanism underlying this association. A central implication of the present results is that tonic dopaminergic properties may act as a foundational constraint on the control system. While pharmacological studies have convincingly demonstrated that transient increases in dopamine can sharpen control signals and reduce distractor influence, such manipulations primarily capture phasic modulation. Our findings instead point toward trait‐like differences in neuromodulatory fidelity that persist across time and contexts. NM‐SD provides a particularly informative marker in this regard. Rather than reflecting average neuromelanin accumulation, which is often interpreted as a proxy for dopaminergic neuron density, the dispersion of the signal captures heterogeneity within the neuronal population (Trujillo et al. 2024). Greater heterogeneity may indicate uneven metabolic burden, localized vulnerability, or variability in neuronal health, all of which could introduce noise into dopaminergic transmission (Trujillo et al. 2024). Because fronto‐striatal circuits rely on dopamine to regulate the signal‐to‐noise ratio of task representations, such variability is likely to propagate into cognitive processing, reducing the precision with which relevant information is amplified and distractors are suppressed (Adelhöfer et al. 2018; Frank 2005, 2025; Yousif et al. 2016).

This framework aligns with computational accounts in which dopamine stabilizes cortical representations by enhancing gain and sharpening attentional gating (Li and Rieckmann 2014; Servan‐Schreiber et al. 1990). When dopaminergic input is reliable, task‐relevant representations can be maintained with high fidelity, minimizing susceptibility to interference (Li and Rieckmann 2014; Servan‐Schreiber et al. 1990). However, if dopaminergic signaling fluctuates due to heterogeneous neuronal integrity, the resulting instability may compromise gating mechanisms, allowing competing information to intrude on response selection (Li and Rieckmann 2014; Servan‐Schreiber et al. 1990). The observed association between elevated NM‐SD and larger Flanker effects is consistent with this interpretation.

Importantly, these results extend beyond the notion that “more dopamine is better.” While NM‐Mean captures overall neuromelanin signal and thus provides a proxy for general dopaminergic integrity, it does not account for how this integrity is distributed across the neuronal population. Instead, they emphasize the functional importance of consistency in neuromodulatory signaling, as indexed by NM‐SD. A uniformly healthy dopaminergic population (low NM‐SD) may support predictable modulation of fronto‐striatal loops, whereas greater heterogeneity (high NM‐SD) could yield irregular control signals—even when overall neuromelanin levels, as reflected in NM‐Mean, appear sufficient.

Notably, whereas NM‐SD was reliably associated with the magnitude of the Flanker effect, it was not associated with conflict adaptation (the Gratton effect). This dissociation is theoretically informative. The Flanker effect primarily indexes the stable efficiency with which individuals suppress distracting information, a process likely supported by tonic dopaminergic properties that regulate baseline gating within fronto‐striatal circuits (Cools 2015; Frank 2005, 2025). In contrast, conflict adaptation reflects rapid, trial‐by‐trial adjustments in control following the detection of conflict—mechanisms commonly linked to medial frontal monitoring systems and transient neuromodulatory dynamics (Botvinick et al. 2004). Such short‐timescale adjustments may depend more strongly on phasic signaling than on the structural integrity of dopaminergic neurons captured by NM‐MRI. Accordingly, spatial heterogeneity in neuromelanin signal may constrain the reliability of sustained interference suppression without necessarily impairing the capacity to flexibly upregulate control after individual high‐conflict events. It is also possible that adaptive control relies on distributed cortical mechanisms capable of compensating for variability in subcortical dopaminergic integrity, thereby preserving moment‐to‐moment adjustments even when baseline signaling is less uniform. Together, this pattern suggests that tonic dopaminergic reliability is more closely tied to stable control efficiency than to dynamic control recalibration, refining current accounts of how distinct dopaminergic properties support separable components of interference control.

We acknowledge an important methodological limitation. Voxel‐wise NM‐SD is sensitive not only to biological variability in dopaminergic integrity but also to differences in in‐plane spatial resolution across participants (0.51–0.66 mm), which arose from subject‐specific adjustments of the field of view (FOV) to maintain scan duration and comply with SAR constraints. As a result, variability in voxel resolution is partially confounded with inter‐individual biological differences and cannot be fully disentangled post hoc. Our pipeline prioritizes the preservation of individual anatomical and signal characteristics by computing voxel‐wise metrics in native space; however, this approach necessarily retains resolution‐related variance alongside biological heterogeneity, rendering NM‐SD a composite measure. Consistent with this, controlling for voxel size reduced the magnitude of the association between NM‐SD and Flanker interference and weakened statistical evidence for its inclusion as a predictor. This attenuation suggests that part of the observed relationship may be influenced by acquisition‐related variance, and that the behavioral relevance of NM‐SD should be interpreted with appropriate caution. At the same time, the direction of the effect remained stable, indicating that NM‐SD may still capture biologically meaningful variability in dopaminergic function, albeit measured with a degree of acquisition‐dependent noise that can modulate effect size and statistical strength. Future studies should aim to minimize this confound at the acquisition stage by fixing voxel resolution across participants. In addition, systematically varying spatial resolution within the same individuals would allow direct quantification of its impact on NM‐SD estimates and further clarify the relative contributions of biological and methodological variance.

A further limitation concerns the biological interpretation of NM‐SD. Although NM‐SD provides a direct quantitative measure of the spatial dispersion of NM‐CNR values within the SN–VTA ROI, its specific biological determinants in healthy populations have not yet been validated. Variability in the relative proportion and spatial distribution of dopaminergic and nondopaminergic tissue within the ROI may contribute to between‐voxel differences in NM‐CNR. However, the extent to which tissue composition determines NM‐SD is itself unknown and cannot be inferred from the present data. Other factors, including individual differences in nigral morphology and microstructure, neuromelanin‐related tissue properties, iron content, ROI geometry, partial‐volume effects, and acquisition‐related characteristics, may also contribute. Thus, the present findings do not establish NM‐SD as a specific biomarker of dopaminergic integrity or signaling reliability. Nevertheless, the absence of a fully established biological interpretation does not preclude the possibility that this measurable property of the NM‐MRI signal carries behaviorally relevant information. Future studies combining NM‐MRI with higher‐resolution anatomical imaging, complementary dopaminergic measures, or histological validation will be required to determine the relative contribution of these factors and establish the biological basis of NM‐SD. A final limitation concerns the influence of subtle head motion on NM‐MRI signal characteristics. The high spatial resolution of NM‐MRI makes the sequence inherently sensitive to sub‐millimeter movement. Because our protocol used online signal averaging (NEX = 2), individual signal averages were not reconstructed or stored separately, limiting retrospective identification and exclusion of motion‐contaminated averages. Although this prevented assessment of motion contamination at the individual‐average level, previous methodological studies using comparable GRE‐MT NM‐MRI acquisition schemes have demonstrated good reliability and reproducibility despite relying on similar averaging approaches (Van Der Pluijm et al. 2021; Wengler et al. 2020), and similar protocols have been successfully implemented on Siemens 3 T Prisma scanners (Liu et al. 2021; Shaff et al. 2024; Wengler et al. 2020). Moreover, all scans underwent strict visual inspection by two independent raters, and datasets showing visible motion artifacts were reacquired when necessary. Although these procedures reduced the likelihood of substantial motion‐related artifacts, we cannot completely exclude the possibility that residual subtle movement contributed to variability in voxel‐wise NM signal estimates. Importantly, however, the direction of motion effects on NM‐SD is not systematic: depending on the pattern of displacement, motion can either increase or decrease heterogeneity. This variability makes it unlikely that motion would produce a consistent association with behavioral performance across participants. Nevertheless, future work using prospective motion correction or multi‐average NM‐MRI sequences would allow more precise quantification and control of motion‐related influences.

Our study underscores the value of moving beyond mean neuromelanin measures (Trujillo et al. 2024). While average signal intensity has been widely used as a marker of dopaminergic neuron density, dispersion metrics may capture functionally meaningful variability that would otherwise remain hidden (Trujillo et al. 2024). Focusing on NM‐SD therefore expands the interpretive potential of neuromelanin imaging and suggests that heterogeneity itself constitutes an important biological signal. Combining NM‐MRI with well‐characterized behavioral paradigms offers a promising avenue for linking neurobiological variability to cognitive phenotypes.

The current findings help bridge two influential but often separate literatures: pharmacological investigations of dopamine and structural imaging approaches to neuromodulatory integrity. Pharmacological studies demonstrate that increasing extracellular dopamine can acutely improve interference control (Gao et al. 2024; Koyun et al. 2025), whereas neuromelanin imaging captures long‐term properties of dopaminergic neurons (Trujillo et al. 2024). Together, these perspectives suggest a multilevel architecture in which trait‐like dopaminergic integrity establishes the operating range within which phasic modulation exerts its effects. One possibility is that individuals with more homogeneous dopaminergic populations are better positioned to benefit from transient dopamine fluctuations because their underlying circuitry is already optimized for signal fidelity. Conversely, when neuronal integrity is uneven, phasic increases may have less predictable consequences, as the neural substrate responsible for implementing control is itself variable. Future studies could combine pharmacological manipulations with neuromelanin‐sensitive imaging to directly test how phasic enhancements interact with trait‐like dopaminergic integrity, providing a more complete understanding of multilevel dopamine contributions to interference control. This integrative view encourages a shift away from thinking about dopamine purely in terms of magnitude and toward considering the structure and reliability of the system through which it acts.

Although the present sample size (*N* = 60) is modest, it exceeds the a priori power estimate and is comparable to or larger than many foundational NM MRI studies in healthy adults (e.g., Hämmerer et al. 2018; Liu et al. 2021; Ye et al. 2021, 2023). NM MRI acquisition imposes substantial logistical constraints, including long scan duration, sensitivity to motion, and hardware driven variability in voxel size, which naturally limit feasible sample sizes even in healthy populations. Moreover, both neuromelanin accumulation and interference control show restricted variability in young adults (Friedman and Miyake 2004; Zucca et al. 2004), likely attenuating observable associations. This restricted range may have contributed to the modest effect sizes observed. Nevertheless, the fact that NM SD—but not NM‐Mean—showed a trend level association with interference control even under these conservative conditions suggests that neuromelanin heterogeneity captures biologically meaningful variability in dopaminergic signaling reliability (Trujillo et al. 2024).

Future studies could also extend this work to better establish causal links and the generalizability of the findings. For example, longitudinal studies tracking changes in NM‐SD alongside cognitive trajectories would help determine whether increasing heterogeneity is associated with emerging control deficits. Additionally, because interference control represents only one facet of executive function, it will be important to examine whether NM‐SD similarly is associated with performance in tasks emphasizing working memory updating, task switching, or reinforcement learning, thereby assessing the broader impact of dopaminergic reliability on cognition.

In summary, the present findings provide tentative evidence that neuromelanin signal variability within the substantia nigra may relate to individual differences in interference control. While NM‐SD showed an association with Flanker performance in the expected direction, this effect was attenuated after controlling for voxel size, and statistical evidence for its inclusion as a predictor was limited. In contrast, NM‐Mean, indexing overall neuromelanin signal, was not associated with behavioral performance, consistent with the notion that overall dopaminergic quantity alone may be insufficient to explain variability in cognitive control. Together, these findings are broadly consistent with a reliability‐based account of dopamine function, in which the consistency of dopaminergic signaling—rather than its absolute level—may contribute to the efficiency of fronto‐striatal control processes, although the present results should be interpreted with caution given potential acquisition‐related confounds.

More generally, these results highlight the importance of considering stable neuromodulatory architecture when explaining individual differences in executive function. Trait‐like properties of the dopamine system may define the boundaries within which flexible control operates, shaping how effectively the brain can prioritize goals while resisting distraction. At the same time, the current findings underscore the need for methodological rigor in isolating biologically meaningful variability from measurement‐related influences. As such, neuromelanin‐derived indices of dopaminergic reliability offer a promising bridge between neurobiology and behavior, opening new avenues for understanding the mechanisms that support adaptive, goal‐directed cognition.

## Author Contributions

All authors had full access to the data, gave final approval for publication, and agreed to be held accountable for the work performed therein. Conceptualization: Lorenza Colzato and Bernhard Hommel. Investigation: Hongchi Zhang and Jimin Yan. Formal analysis: Shuhui Lyu, Jiaqi Gao, and Kangcheng Wang. Visualization: Shuhui Lyu. Writing – original draft: Shuhui Lyu, Lorenza Colzato, and Bernhard Hommel. Writing – reviewing and editing: all authors. Supervision: Bernhard Hommel. Funding acquisition: Bernhard Hommel.

## Funding

The “One case, one policy” grant from Shandong Province (China) awarded to B.H.

## Ethics Statement

All participants provided written informed consent and received a financial reimbursement for their participation in the study. The ethics committee of Shandong Normal University approved this study.

## Consent

The authors have nothing to report.

## Conflicts of Interest

The authors declare no conflicts of interest.

## Supporting information


**Table S1:** Comparison between included participants and all excluded participants.
**Table S2:** Comparison between included participants and participants excluded due to incomplete neuromelanin MRI data.
**Table S3:** Comparison of zero‐order and covariate‐adjusted associations between NM metrics and behavioral performance.
**Table S4:** FDR‐corrected associations between neuromelanin metrics and cognitive control measures.
**Figure S1:** Participant selection and exclusion flow diagram.

## Data Availability

The data that support the findings of this study are openly available in open science framework at https://osf.io/q4fvk/overview.

## Endnotes

^1^
The first session also included EEG measurements for purposes unrelated to the present study. In the second session, participants also carried out the Flanker task in the fMRI. However, given that no significant Flanker effect was obtained in this task (for reasons that might reflect the specific characteristics of the fMRI environment: Hommel et al. 2012), we will not consider task data from this session.
