# Supplementary material for: Variability, but Not Concentration, of Neuromelanin in the Substantia Nigra Is Associated With Individual Differences in Interference Control
Source: Hum Brain Mapp. 2026 Nov 9;47(16):e70663. doi: 10.1002/hbm.70663 (PMC13748931; doi:10.1002/hbm.70663)
Supplement: Supplementary file 1 [file HBM-47-e70663-s001.docx]

Supplementary Material

**Variability, but not Concentration, of Neuromelanin in the Substantia Nigra is Associated with Individual Differences in Interference Control**

Shuhui Lyu, Hongchi Zhang, Jimin Yan, Jiaqi Gao, Kangcheng Wang, Lorenza Colzato, Bernhard Hommel

**Table S1.**

*Comparison between included participants and all excluded participants*

| Measure | Included  (n=60) | | Excluded  (n=22) | | Statistic | *p* | Effect size |
| --- | --- | --- | --- | --- | --- | --- | --- |
|  | Mean | SD | Mean | SD |  |  |  |
| Sex (M/F) | 25/35 |  | 13/9 |  | Fisher's exact | 0.213 | OR=0.49 |
| Age (years) | 20.4 | 1.1 | 20.3 | 1.1 | *t*_(35.7)_ = 0.52 | 0.605 | d=0.13 |
| FE (ms) | 59.5 | 28.5 | 49.0 | 36.9 | *t*_(30.7)_ = 1.21 | 0.237 | d=0.34 |
| Flanker accuracy (%) | 87.2 | 7.5 | 84.2 | 13.3 | *t*_(26.0)_ = 1.02 | 0.319 | d=0.33 |

Note. All continuous-variable comparisons used Welch's t-test; degrees of freedom were approximated via the Welch-Satterthwaite equation. Sex was compared using Fisher's exact test. FE: Reaction Time of Flanker effect in EEG session; M: Male; F: Female

**Table S2.**

*Comparison between included participants and participants excluded due to incomplete neuromelanin MRI data.*

| Measure | Included  (n=60) | | Excluded  (n=20) | | Statistic | *p* | Effect size |
| --- | --- | --- | --- | --- | --- | --- | --- |
|  | Mean | SD | Mean | SD |  |  |  |
| Sex (M/F) | 25/35 |  | 13/7 |  | Fisher's exact | 0.120 | OR=0.38 |
| Age (years) | 20.4 | 1.1 | 20.4 | 1.1 | *t*_(30.8)_ = 0.23 | 0.819 | d=0.06 |
| FE (ms) | 59.5 | 28.5 | 49.0 | 38.5 | *t*_(26.3)_ = 1.12 | 0.274 | d=0.34 |
| Flanker accuracy (%) | 87.2 | 7.5 | 86.8 | 7.5 | *t*_(32.4)_ = 0.20 | 0.844 | d=0.05 |

Note. All continuous-variable comparisons used Welch's t-test; degrees of freedom were approximated via the Welch-Satterthwaite equation. Sex was compared using Fisher's exact test. FE: Reaction Time of Flanker effect in EEG session; M: Male; F: Female

**Table S3.**

*Comparison of zero-order and covariate-adjusted associations between NM metrics and behavioral performance*

| Analysis | Zero-order | | Voxel size adjusted | | Voxel Count adjusted | |
| --- | --- | --- | --- | --- | --- | --- |
|  | *r* | *p* | *r* | *p* | *r* | *p* |
| NM-SD → FE | 0.321 | 0.012 | 0.189 | 0.154 | 0.086 | 0.516 |
| NM-Mean → FE | 0.060 | 0.649 | 0.045 | 0.724 | 0.075 | 0.553 |
| NM-SD → GE | 0.137 | 0.302 | –0.020 | 0.882 | –0.083 | 0.535 |
| NM-Mean → GE | –0.161 | 0.219 | –0.178 | 0.179 | –0.158 | 0.237 |

Note. All adjusted analyses used the Freedman–Lane permutation procedure (5,000 iterations). FE: Flanker effect. GE: Gratton effect

**Table S4.**

*FDR-corrected associations between neuromelanin metrics and cognitive control measures*

Panel A. Zero order associations

| Predictor | Outcome | *r* | *p* | FDR adjusted *p* |
| --- | --- | --- | --- | --- |
| NM-SD | FE | 0.321 | 0.012 | 0.048^*^ |
| NM-SD | GE | 0.137 | 0.302 | 0.403 |
| NM-Mean | FE | 0.06 | 0.649 | 0.649 |
| NM-Mean | GE | -0.161 | 0.219 | 0.403 |

Panel B. Voxel size adjusted associations

| Predictor | Outcome | *r* | *p* | FDR adjusted *p* |
| --- | --- | --- | --- | --- |
| NM-SD | FE | 0.189 | 0.154 | 0.358 |
| NM-SD | GE | -0.02 | 0.882 | 0.882 |
| NM-Mean | FE | 0.045 | 0.724 | 0.882 |
| NM-Mean | GE | -0.178 | 0.179 | 0.358 |

Note. Benjamini–Hochberg false discovery rate correction was applied separately to each predefined analysis family (four tests per family). All p-values were two-tailed permutation p-values obtained from 5,000 permutations. * Indicates associations surviving FDR correction (FDR adjusted *p* < 0.05). Panel B presents voxel size adjusted sensitivity analyses using the Freedman–Lane permutation procedure, with voxel size included as a continuous covariate. FE: Flanker effect. GE: Gratton effect

**Fig. S1.**

*Participant selection and exclusion flow diagram*
